# Elastic Fibre Proteins in Elastogenesis and Wound Healing

**DOI:** 10.3390/ijms23084087

**Published:** 2022-04-07

**Authors:** Xinyang Zhang, Yasmene F. Alanazi, Thomas A. Jowitt, Alan M. Roseman, Clair Baldock

**Affiliations:** 1 Wellcome Centre for Cell-Matrix Research, Faculty of Biology, Medicine and Health, University of Manchester, Manchester Academic Health Science Centre, Manchester M13 9PT, UK; xinyang.zhang-2@postgrad.manchester.ac.uk (X.Z.); thomas.a.jowitt@manchester.ac.uk (T.A.J.); 2 School of Biological Sciences, Faculty of Biology, Medicine and Health, University of Manchester, Manchester Academic Health Science Centre, Manchester M13 9PT, UK; alan.roseman@manchester.ac.uk; 3 Department of Biochemistry, Faculty of Science, University of Tabuk, Tabuk 71491, Saudi Arabia; yalenazi@ut.edu.sa

**Keywords:** fibrillin-1, tropoelastin, latent TGFβ binding protein (LTBP)-4, fibulin-4, fibulin-5

## Abstract

As essential components of our connective tissues, elastic fibres give tissues such as major blood vessels, skin and the lungs their elasticity. Their formation is complex and co-ordinately regulated by multiple factors. In this review, we describe key players in elastogenesis: fibrillin-1, tropoelastin, latent TGFβ binding protein-4, and fibulin-4 and -5. We summarise their roles in elastogenesis, discuss the effect of their mutations on relevant diseases, and describe their interactions involved in forming the elastic fibre network. Moreover, we look into their roles in wound repair for a better understanding of their potential application in tissue regeneration.

## 1. Introduction

Elastic fibres endow tissues and organs with elasticity and extendibility in response to mechanical forces. Aberrant formation and destruction of elastic fibres leads to many diseases, such as Marfan syndrome (MFS) [1], cutis laxa and aneurysms [2]. Elastic fibres are formed predominantly from elastin and fibrillin microfibrils [3]. Elastic fibre proteins guide and facilitate elastogenesis, where tropoelastin globules are deposited on a fibrillin microfibril scaffold, a process which is facilitated by fibulin-4 and -5 and latent TGFβ-binding protein (LTBP)-4. In addition to elastogenesis, elastic fibre proteins have been implicated in wound healing: for instance, in keloid disease and hypertrophic scarring, disorganised and reduced elastin and fibrillin has been observed [4,5]. Furthermore, elastic fibre proteins are important players in regulating TGFβ signalling [6] and integrin-mediated cell attachment and spreading, which can further contribute to wound healing. Thus, this review focuses on the elastic fibre proteins tropoelastin, fibrillin-1, LTBP4, fibulin-4 and -5, and discusses their roles in elastogenesis and wound repair.

## 2. Elastic Fibre Proteins and Their Roles in Elastogenesis

### 2.1. Fibrillin-1

**Figure 1:**
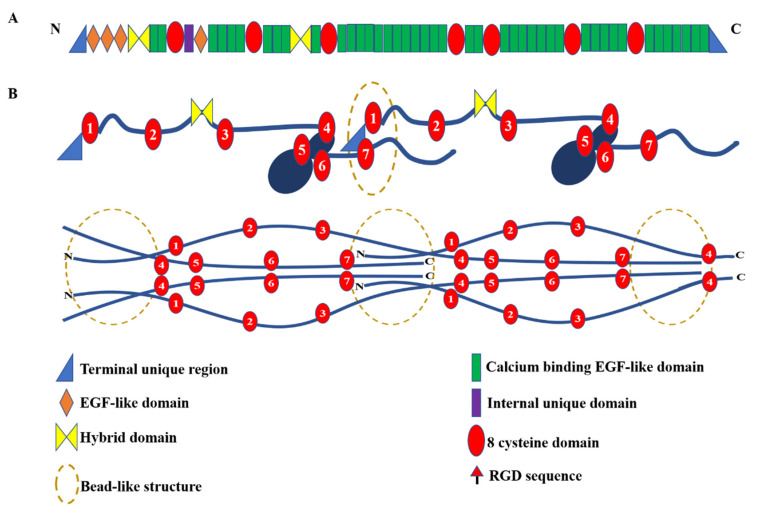
Schematic diagram of fibrillin-1 domain structure and microfibril organisation. (**A**) Fibrillin-1 is a modular multi-domain protein predominantly composed of calcium binding EGF-like domains, TB and hybrid domains. Fibrillin-1 has a unique N-terminal (FUN) and C-terminal domain, and the internal unique domain is proline-rich; (**B**) The pleated model (**upper**) and staggered model (**lower**) of fibrillin microfibril organisation. In the pleated model, the fibrillin-1 monomer is compressed and folded within one interbead repeat (57 nm period). In the staggered model, each fibrillin-1 monomer is staggered in a head-to-tail pattern spanning two or three interbead repeats. The TB or 8-cysteine domains are numbered.

In humans, the fibrillin family is composed of three highly conserved proteins, fibrillin-1, -2 and -3, all of which are engaged in the formation of microfibrils. Fibrillin-2 and -3 are mainly expressed in fetal tissues, while fibrillin-1 is continuously expressed throughout adulthood in tissues such as the heart, aorta, lung, nervous system and skin [7,8]. Mutations in the FBN1 gene, which encodes fibrillin-1, are associated with MFS, isolated autosomal dominant ectopia lentis 1, mitral valve-aorta-skeleton-skin (MASS) syndrome [9], Weill–Marchesani syndrome (WMS) [10], stiff skin syndrome [11], acromicric and geleophysic dysplasias [12] and Marfanoid-progeroid-lipodystrophy syndrome [13]. Human fibrillin-1 is composed of 2781 amino acids and contains multiple domains, the majority of which are calcium binding EGF-like (cbEGF) domains [14,15]. Other domains are the fibrillin unique N-terminal (FUN) region, 8-cysteine domains (also known as TGFβ binding-like or TB domains), hybrid domains, a proline-rich region and a C-terminal region, as shown in Figure 1 [3,16]. In vitro experiments have shown that fibrillin-1 interacts with itself, leading to microfibril assembly [17,18,19], and interacts with fibrillin-2 [17], heparan sulphate [20,21,22], microfibril-associated glycoproteins [23], fibronectin [24] and other elastic fibre proteins discussed later in this review, to form elastic fibres.

The importance and function of fibrillin in vivo has been probed using a range of mouse models. In mgΔ/mgΔ mice, in which exons 19–24 of FBN1 are deleted, no gross phenotypic abnormalities were observed at birth, but mice died suddenly around three weeks of age, and were characterised as vascular compromised, with aneurysmal dilatation, focal fragmentation of elastic fibres and accumulation of the amorphous matrix observed [25]. Depending on genetic background, heterozygous mice had a normal lifespan, but showed some classic MFS phenotypes, including pulmonary alterations and disruption or degradation of the elastic fibres [26]. Disorganised elastic fibres were observed in the cornea of the fibrillin-1 mgΔ heterozygous mice by electron microscopy and X-ray scattering [27]. In addition, fibrillin-1 MFS mouse models with point mutations, domain deletion or truncations have also been generated to determine the role of fibrillin-1 in elastic-fibre-associated diseases (for review, see [28,29]). In a model of WMS, the WMΔ mice with in-frame deletion of exons 9–11 in FBN1 had thickened, less elastic skin and altered ultrastructure of fibrillin microfibrils [30].

Despite our knowledge of the tissue role of fibrillin microfibrils, how the ~150 nm long fibrillin monomers are organised into microfibrils with a periodicity of ~57 nm is still not fully resolved. When visualised by electron microscopy, microfibrils have a “beads-on-a string” appearance [3]. Two models have been suggested for the packing of fibrillin molecules within microfibrils based on a range of data, including small angle X-ray scattering (SAXS), electron tomography, antibody mapping and X-ray crystallography [31,32,33,34]. In the pleated model, the N- and C-termini are overlapped within the bead, and the remaining domains are arranged within the interbead so one fibrillin monomer spans a single 57 nm microfibril repeat (Figure 1B). In the linear model, the termini are also overlapped within the bead, but the fibrillin monomers are staggered in the microfibrils, and could span two or more interbead repeats (Figure 1B).

### 2.2. Tropoelastin

**Figure 2:**
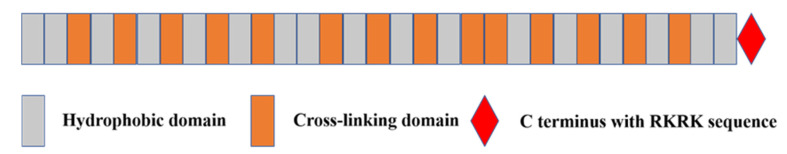
Domain structure of tropoelastin. Valine, proline and glycine-rich hydrophobic domains are involved in the self-assembly or coacervation of tropoelastin. Hydrophilic domains, rich in lysine, alanine and proline residues are arranged alternately between hydrophobic domains and contribute to the cross-linking of tropoelastin. The C-terminal RKRK motif binds with integrins to regulate cell adhesion and interacts with microfibrils to facilitate elastic fibre assembly.

Tropoelastin is the soluble precursor of elastin and is encoded by the ELN gene. The most common splice form of human tropoelastin is ~60 kDa, containing cross-linking domains rich in lysine residues and hydrophobic domains rich in proline and glycine residues, as shown in Figure 2 [35]. Tropoelastin is secreted to the cell surface by elastogenic cells, and then undergoes rapid spontaneous self-assembly or coacervation to form spherical structures under physiological conditions via specific interaction sites on its hydrophobic domains [36]. These structures are stabilised by cross-linking via its lysine residues mediated by lysyl oxidase to further form tetrafunctional desmosine cross-links [37]. Elastin globules are then deposition onto fibrillin microfibrils with the assistance of elastic-fibre-associated proteins to form elastic fibres. This is facilitated by specific functional regions on the microfibrils, and is supported by the elastic fibre proteins fibulin-4, fibulin-5 and LTBP4, which will be described within this review. This complex and orchestrated process has been described and reviewed extensively elsewhere [38,39]. The expression of tropoelastin is initiated and increases rapidly at the late stage of fetal development [40], whereas there is hardly any de novo synthesised tropoelastin in adulthood. Despite its limited synthesis time window, elastin is stable once deposited, having an estimated half-life of several decades and potentially up to 70 years [41].

The 3D structure of tropoelastin was first analysed by small-angle neutron scattering (SANS) and SAXS, and showed that the tropoelastin molecule is asymmetric with a “head-like” N-terminal region and a “foot-like” C-terminal region. An extended coil region, a flexible hinge and a bridge region are located between the N- and C-terminal regions [42]. More recently, using replica exchange molecular dynamics simulations (REMD), the fully atomistic molecular structure of human tropoelastin was modelled and found to have common structural features and similar dimensions to the SAXS tropoelastin model [43]. Discrepancies in local structure observed between these two models reflect the dynamic properties of tropoelastin. Notably, there are 13 transcript variants of tropoelastin displayed in the NCBI, and results from several studies by nuclear magnetic resonance (NMR) and SAXS [44,45] suggest that different tropoelastin isoforms from different transcript variants may have remarkable effects on the structure of tropoelastin. It may be that the tropoelastin isoforms express in a tissue- and/or development-specific manner to further influence the formation or properties of elastic fibres.

Mutations in tropoelastin can result in cutis laxa (CL) and supravalvular aortic stenosis [9]. In Williams syndrome (WS) patients, a 500 kb region at 7q11.23 containing ELN and other genes is deleted [9], suggesting the important role of tropoelastin in the aetiology of WS. The relationships between polymorphisms of tropoelastin and other diseases have also been studied, such as aortic dissection [46] and abdominal aortic aneurysm [47].

### 2.3. Latent TGFβ Binding Protein (LTBP)-4

**Figure 3:**
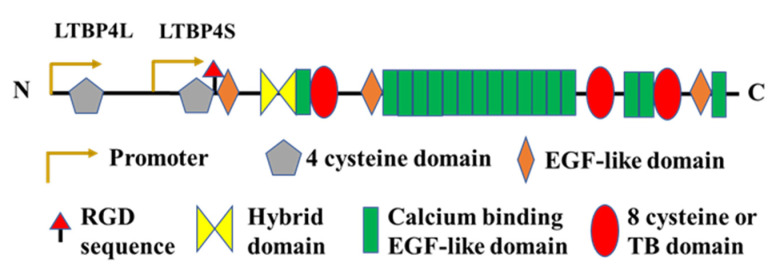
Domain arrangement of LTBP4. The domain structure of LTBP4L and LTBP4S are both characterised by multiple calcium-binding EGF-like domains and 8-cysteine domains, but the transcription of LTBP4L and LTBP4S are initiated by independent promoters, resulting in tissue-specific expression patterns of LTBP4L and LTBP4S.

The LTBPs have similar domain composition to fibrillin and are therefore members of the fibrillin superfamily. In humans, there are four LTBP isoforms, namely LTBP1-4. The LTBPs were named due to their role in the latency of TGFβ, where the formation of a covalent disulphide bond between LTBP1, -3 and -4 with the propeptide of TGFβ results in the formation of a large latent TGFβ complex, an important regulator of TGFβ signalling. Both LTBP2 and LTBP4 are involved in elastic fibre formation, but here we focus on LTBP4 due to its essential role in elastogenesis, as evidenced by the pathology observed in humans and mice with mutations in LTBP4 [48,49,50]. LTBP4 is an extracellular glycoprotein encoded by the LTBP4 gene, and has the highest expression in the heart, small intestine and uterus, followed by the ovary, adrenal gland and aorta [51]. There are at least four transcripts of LTBP4 produced by alternative splicing, including LTBP4L, LTBP4S, LTBP4Δ2E and LTBP4ΔE, of which LTBP4L and LTBP4S are the major isoforms with distinct functions and tissue-specific expression [48,52]. LTBP4 is also a genetic modifier of Duchenne muscular dystrophy (DMD), where polymorphisms in LTBP4 have been linked to the age at loss of ambulation in DMD patients [53,54]. The domain structure of LTBP4 is homologous to fibrillin-1 with 8-cysteine domains and EGF-like domains, the majority of which also bind calcium, as shown in Figure 3.

Mutations in LTBP4 are associated with an inherited connective tissue disease, autosomal recessive cutis laxa type 1C (ARCLIC) in humans [55], which is recapitulated by an ARCLIC-like phenotype in LTBP4 deficient mice [48]. ARCLIC patients have CL in addition to pulmonary, intestinal and facial abnormalities. Immunohistological and electron microscopy studies on both skin and lung sections from patients with either homozygous or heterozygous LTBP4 mutations showed abnormal elastic fibres. Fragmented elastic fibres were observed in the deep dermis of the skin, while in the papillary dermis, elastic fibres were diminished [56]. The lung sections showed enlarged air sacs with fragmented elastic fibres and other areas with collapsed air sacs. LTBP4S-deficient mice showed similarly abnormal ultrastructure of elastic fibres in their lungs. Knock-down of LTBP4 in human dermal fibroblast cells and knock-out of LTBP4S in mice resulted in a punctate deposition of elastin, but addition of recombinant LTBP4S enhanced elastic fibre assembly [50]. LTBP4 is facilitated by members of the fibulin family in elastogenesis in an LTBP4L- or LTBP4S-isoform-specific manner [49,50].

### 2.4. Fibulin-4 and Fibulin-5

**Figure 4:**
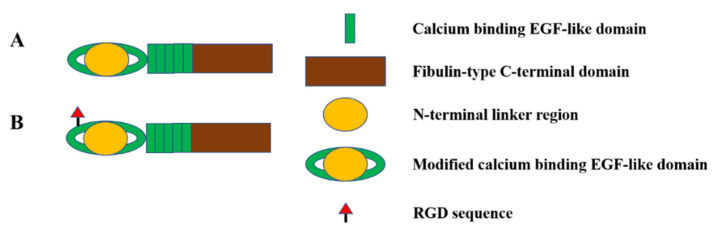
Domain structure of fibulin-4 and fibulin-5. Both fibulin-4 (**A**) and fibulin-5 (**B**) are composed of cbEGF domains and a fibulin-type C-terminal domain. In addition, fibulin-5 has an integrin-binding RGD site.

The fibulin family contains long fibulins (fibulin-1 and -2), short fibulins (fibulin-3, -4, -5 and -7) and hemicentins (fibulin-6 and -8) [57]. Among them, fibulin-4 and fibulin-5 have discrete and essential roles in elastic fibre formation [58]. Fibulin-4 and fibulin-5, encoded by the FBLN4 and FBLN5 genes, are characterised by cbEGF domains and a C-terminal fibulin domain, as shown in Figure 4. Mutations in FBLN4 result in a spectrum of phenotypes, including CL, deformation or occlusion of elastic arteries, aortic aneurysm and arachnodactyly [59,60,61]. These findings show that fibulin-4 plays an indispensable role in elastogenesis. Fibulin-4 regulates the self-assembly of elastin, which has been shown in vitro with an elastin-like polypeptide [62], and together with fibrillin regulates elastin deposition onto microfibrils [58]. Fibulin-4 directly binds the cross-linking enzyme lysyl oxidase, and forms a ternary complex by further interacting with tropoelastin, facilitating the cross-linking of tropoelastin [58]. LTBP4 also binds fibulin-4 in an isoform-specific manner [48,49]. The deposition of fibulin-4 is normal in mice that only express the long isoform of LTBP4 but is deficient in LTBP4^−/−^ null mice [48]. Furthermore, the addition of fibulin-4 to wildtype and LTBP4S^−/−^ fibroblasts showed a normal linear deposition of the exogenous fibulin-4, while cell cultures from LTBP4^−/−^ showed a scattered and globular deposition of recombinant fibulin-4 [48], suggesting a functional interaction with LTBP4L is required for correct fibulin-4 deposition. Fibulin-4 has also been suggested to induce a stable conformational and functional change in LTBP4L, which promotes tropoelastin deposition onto the elongated LTBP4L [63].

**Figure 5:**
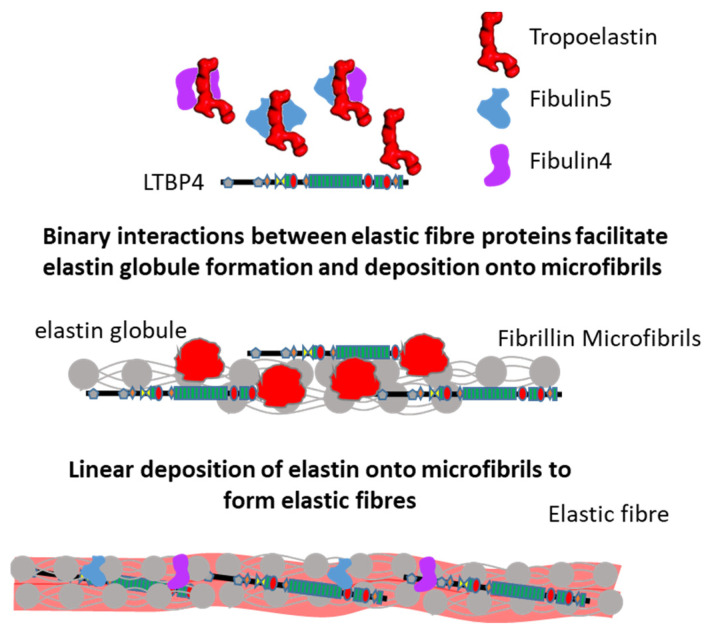
Model for elastic fibre assembly. Both fibulin-4 and fibulin-5 and their complexes with tropoelastin bind to LTBP4, and tropoelastin can also bind directly to LTBP4. These complexes mediate the deposition of elastin onto a fibrillin microfibril scaffold, supported by molecular interactions between fibrillin, tropoelastin, LTBP4, fibulin-4 and -5, as detailed in Table 1.

Fibulin-5 is predominantly expressed in the heart, ovary and colon [64], and has been linked to CL [65,66]. In 1.7% of age-related macular degeneration (AMD) patients, missense mutations in fibulin-5 were found [67,68], and structural analysis of CL and AMD mutations revealed that the mutations in fibulin-5 altered the structure, which may contribute to AMD and CL [69]. These pathologies are linked to defective elastic fibre assembly, suggesting an important role for fibulin-5 in elastogenesis. Indeed, fibulin-5 was found to affect the self-assembly and coacervate maturation of an elastin-like polypeptide in vitro [62]. Using sandwich binding assays, fibulin-5 was found to act as an adapter mediating the binding of fibrillin-1 to tropoelastin [70]. Furthermore, after fibulin-5 knockdown elastin globules with limited association to microfibrils were observed in rat fetal lung fibroblasts, indicating their necessary role in elastin globule deposition onto microfibrils [58]. Knockdown of LTBP4 in fibroblast cultures prevented the deposition of both elastin and fibulin-5, and the addition of fibulin-5 did not rescue this effect, whereas the addition of LTBP4 restored the deposition of elastin and fibulin-5 [50]. Together, these studies show that the deposition of elastin–fibulin complexes onto the microfibril scaffold requires LTBP4 (Figure 5), and that these processes are underpinned by numerous molecular interactions, as described in the following section.

### 2.5. Interactions Supporting Elastic Fibre Assembly

Multiple studies have demonstrated that fibrillin-1, tropoelastin, LTBP4, fibulin-4 and -5 interact in order to implement their function in elastogenesis, as shown in Table 1. Fibulin-5 interacts with tropoelastin via binding sites throughout the fibulin-5 molecule [58,71,72], and mutations in fibulin-5 can either reduce or increase its affinity for tropoelastin [73,74]. Similarly, fibulin-4 strongly interacts with tropoelastin in the presence of Ca^2+^, and also in solution, as evidenced by co-immunoprecipitation [75]. Comparatively, tropoelastin binds with higher affinity to fibulin-5 than fibulin-4, based on SPR analysis [58]. These interactions are thought to facilitate the cross-linking of tropoelastin and subsequent deposition of tropoelastin onto microfibrils. In addition, fibulin-4 and fibulin-5 can either self-associate [62,76] or interact with each other [58], but whether this has a role in elastogenesis remains unclear.

Fibulin-4 and -5 also interact with LTBP4 and fibrillin-1, thus promoting the deposition of tropoelastin–fibulin complexes onto microfibrils. In particular, the C-terminal domain of fibulin-5 interacts with an N-terminal region of LTBP4 [50]. The interaction between fibulin-4 and LTBP4 is also mediated via an N-terminal region, with both long and short isoforms of LTBP4 binding to fibulin-4, but LTBP4L binds fibulin-4 more tightly than LTBP4S [48] via a central region of fibulin-4 [63]. Interestingly, our group recently found that tropoelastin can directly bind the C-terminal region of LTBP4 via binding studies using Biolayer interferometry [77], but the function of the LTBP4–tropoelastin interaction in elastic fibre assembly remains to be further explored.

Consistent with a role in facilitating the deposition of tropoelastin–fibulin complexes onto microfibrils, fibulin-4 and -5 both bind with high affinity to the N-terminal half of fibrillin-1 [71], and the N-terminal hybrid1 domain in fibrillin is required for this interaction [70]. A CL causing S227P mutant in fibulin-5 impaired its interaction with fibrillin-1, as observed by immunostaining in vitro [73], and CL mutations A397T, E57K and E126K in fibulin-4 resulted in impaired binding to fibrillin-1 [78].

In addition, fibrillin-1 also interacts with LTBP4 via the N-terminal hybrid1 domain to incorporate LTBP4 into microfibrils, since deletion of this domain abolishes the binding of fibrillin-1 to LTBP4, and an N164S mutation reduced binding to LTBP4 [79]. Fibrillin and tropoelastin also interact directly, with the central sequence of fibrillin-1 interacting with tropoelastin [80]. As these elastic fibre proteins can all form binary interactions in vitro (detailed in Table 1), what remains to be elucidated is the hierarchy and order of interactions required for effective elastogenesis in vivo.

**Table 1:** Interactions and functions of elastic fibre proteins.

Interaction	Function
Fibrillin-1–fibulin-4 [58,70,71,78]	Tropoelastin cross-linking and deposition onto microfibrils
Fibrillin-1–fibulin-5 [58,70,71,73]	
Tropoelastin–fibulin-4 [58,75]	
Tropoelastin–fibulin-5 [58,71,72,73,74]	
Tropoelastin–fibrillin-1 [80]	Tropoelastin deposition and elastic fibre formation
Fibrillin-1–LTBP4 [79]	Deposition and sequestering of latent TGFβ in the extracellular matrix
Fibulin-4–fibulin-5 [58]	Unknown: Might contribute after initial elastin cross-linking
Tropoelastin–LTBP4 [77]	Unknown: Might contribute to elastic fibre formation
LTBP4–fibulin-5 [50]	Deposition of fibulin-5 and tropoelastin on microfibrils
LTBP4–fibulin-4 [48,63]	Conformational switch of LTBP4 structure, deposition of tropoelastin onto the elongated LTBP4, and deposition of fibulin-4 on microfibrils

## 3. The Role of Elastic Fibre Proteins in Wound Repair

In addition to their role in elastogenesis, there is increasing evidence demonstrating the importance of these elastic fibre proteins in wound repair. In a periodontal disease model, fibrillin-1 expression was strongly elevated at the beginning of the destruction of periodontal tissue, but decreased with wound healing [81]. This decrease in fibrillin-1 expression during wound healing has been associated with the differentiation of fibroblasts to myofibroblasts in dental pulp healing [82]. Overexpression of fibulin-5 in a dermal ulcer model showed that fibulin-5 expression facilitates wound healing in vivo [83]. Numerous reports have demonstrated the role of tropoelastin in the inflammation and proliferation stages of wound healing; for example, tropoelastin induced transient expression of chemokines, which are necessary for tissue recovery [84]. Elastic fibre proteins are also important for the extracellular regulation of TGFβ, an important mediator of wound healing [85]. Thus, in the following section, we review the role of elastic fibre proteins in TGFβ sequestration and activation.

### 3.1. Elastic Fibre Proteins and TGFβ Signalling

TGFβ is secreted as a large latent complex (LLC) covalently linked to members of the LTBP family. A disulphide bond is formed between LTBP1, 3 and 4 and the TGFβ pro-domain (latency-associated peptide (LAP)), and the LLC then deposits into the extracellular matrix via the interactions between LTBPs and fibrillin and fibronectin [86]. LTBPs influence TGFβ signalling by at least two mechanisms: promoting effective secretion of latent TGFβ from cells [87,88], and the localisation of latent TGFβ in the matrix [86]. LTBP4 interacts with different isoforms of TGFβ (TGFβ1, β2, β3), and two different LTBP4 SNPs enhance and reduce TGFβ signalling, respectively [89]. Co-immunoprecipitation showed an interaction between LTBP4 and the TGFβ receptor 2, and knock-down of LTBP4 reduced the expression of TGFβ receptor 2 and signalling [90]. Lu et al. found that knock-down of LTBP4 in systemic scleroderma skin fibroblasts reduced the extracellular level of TGFβ and the downstream targets of TGFβ signalling [91].

Integrins are activators of TGFβ by binding to and unfolding LAP to release mature TGFβ from the latent complex to enable TGFβ receptor binding [92]. Binding of LAP to LTBP is required to provide resistance to the pulling force [93]. Recently, Campbell et al. also showed by cryo-EM that αvβ8 could activate latent TGFβ without releasing mature TGFβ from the latent complex [94]. Fibrillin-1 has been linked to the regulation and bioavailability of TGFβ in the extracellular matrix via direct interaction with LTBP1 and LTBP4 and via stabilising the LLC [95,96]. Although the mechanisms are not fully elucidated, many studies support a role for fibrillin-1 in TGFβ sequestration. For example, fibrillin-1 mutations are associated with MFS, which is linked to an increase in TGFβ activation in connective tissues [96], and osteoblasts from Fbn1^−/−^ mice have more activated TGFβ [97]. In addition, fibrillin-1 was found to influence pSmad2-dependent TGFβ signalling via regulating the expression of miR-503 in fibroblasts [98].

In fibulin-4-deficient aortic smooth muscle cells, elevated TGFβ signalling was observed due to increased levels of TGFβ2 [99]. Interestingly, Burger et al. found that in vascular smooth muscle cells, reduced fibulin-4 expression enhanced the activation of TGFβ, but there was no change in TGFβ signalling when fibulin-4 was absent [100]. Fibulin-5 expression is reported to be regulated by TGFβ in fibroblasts and mammary epithelial cells [101,102,103,104], and fibulin-5 overexpression in 3T3-L1 cells elevated the TGFβ-stimulated activation of ERK1/ERK2 and p38 MAPK [104], indicating that fibulin-5 is also involved in TGFβ signalling.

### 3.2. Role of Elastic Fibre Proteins Supporting Integrin-Mediated Cell Adhesion

In addition to their role in supporting TGFβ secretion and activation, elastic fibre proteins support integrin-mediated cell adhesion. Integrins αvβ3, α5β1, αvβ6, α8β1, αvβ6, αvβ1 and αvβ5 can bind to the TB4 domain of fibrillin-1 via an RGD sequence in cell-based assays or protein–protein interaction analyses [33,105,106,107,108]. In addition, fibrillin-1 was found to influence integrin-mediated focal adhesion formation by regulating the expressions of miR-612 and miR-3185 in fibroblasts [98]. Bax et al. found that the C-terminal GRKRK sequence in tropoelastin supports cell adhesion via interaction with αvβ3 [109]. The same group also found that αvβ5 can interact with the central region of tropoelastin to mediate cell adhesion [110], and Bochicchio et al. found that domains 12 to 16 of tropoelastin can interact with integrins αv and α5β1, thus promoting cell spreading and attachment [111]. Modelling data linked these findings to show that different regions on tropoelastin bind to multiple sites on integrin αvβ3 to co-operatively support signalling [112]. Fibulin-5 binds human smooth muscle cells (SMC) via integrins α5β1 and α4β1, and influences SMC proliferation and migration, but does not support the activation of receptor tyrosine kinases [113]. In addition, Furie et al. found that fibulin-5 binds to keloid-derived fibroblast-like cells (FLC) and regulates FLC adhesion and proliferation through integrin β1 [114].

**Figure 6:**
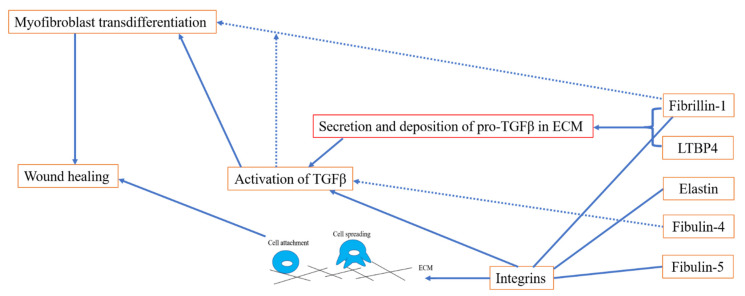
Diagram of elastic fibre proteins in wound healing. Deposition and sequestration of pro-TGFβ in the ECM is crucial for the proper regulation of TGFβ via fibrillin-1 and LTBP4. In addition, fibrillin-1 may be involved in myofibroblast transdifferentiation in a TGFβ-dependent way. Fibrillin-1, tropoelastin and fibulin-5 are also involved in the process of wound repair by regulating cell adhesion via integrins.

Collectively, elastic fibre proteins play an important role in wound healing via regulating the deposition and activation of TGFβ and supporting integrin-mediated cell adhesion, as shown in Figure 6.

## 4. Perspectives

Although the roles of fibrillin-1, tropoelastin, LTBP4, fibulin-4 and -5 in elastogenesis have been widely studied, many scientific questions remain to be elucidated. Deciphering whether interactions between LTBP4 and tropoelastin support either elastogenesis or LTBP4-mediated TGFβ signalling in wound healing, and the role fibrillin plays in these processes, are of great significance in tissue regeneration and elastic fibre diseases. Additionally, deciphering the order and hierarchy of interactions between all the elastic fibre proteins is important to understand the sequence of events and molecular requirements for elastogenesis. Considering the importance of myofibroblasts in wound healing, exploring the detailed molecular mechanisms of how elastic fibre proteins influence myofibroblast differentiation may provide opportunities for novel therapeutics for wound repair. For instance, elucidating whether changes in the expression of elastic fibre proteins or dysfunction of elastic fibres in scar tissue alters their biomechanical properties, such as contractility, to negatively influence myofibroblast differentiation would be an important future research direction.
